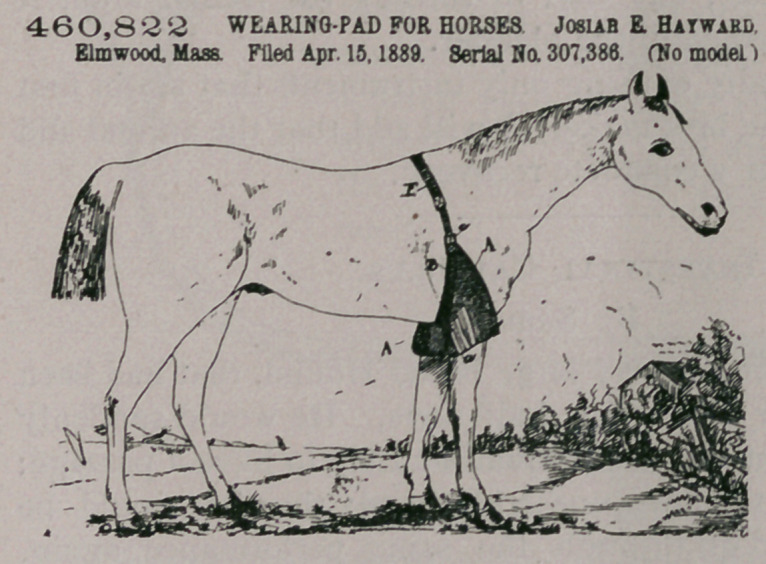# Recent Patents

**Published:** 1891-11

**Authors:** 


					﻿RECENT PATENTS
RELATING TO
VETERINARY MEDICINE AND ANIMAL INDUSTRY.
Issued by U. S. Patent Office since August, 1891.
Claim.—A removable
attachment for horse-
shoes consisting of two
separate sections a al,
formed to fit the inner
edges of a horseshoe, ad-
justable heel and toe
calks adapted to pass
through said sections
and engage the outer
face of the shoe to pre-
vent the attachment be-
coming loose upon the
same, and adjusting-
screws working in said
sections on opposite sides of their centres of length to clamp the attachment in
place when expended and release it when contracted, substantially as set forth.
Claim.—The combin-
ation of the u-shaped
body A, formed with the
longitudinal recesses P
and recessed at its outer
end to form the seat a,
the removable blade C,
curved to conform to the
curved outer end of the
body A and having the
curved cutting-edge, the
frame-pieces E, secured to the inner ends of the body A, the blade D, sliding in the
recesses B, the pivoted hand-levers, and the straight connecting-links G, pivotally
connecting the inner end of the hand-levers with the lower end of the sliding
blade D, substantially as set forth.
Claim.—In combina-
tion with a horseshoe
having the dovetail slot
and the narrow flange at
the forward end, the ex-
tension A, having the
narrow flange m ml, and
the dovetail tongue D,
with corresponding per-
forations in the dovetail
tongue and the dovetail
slot for the reception of
the screw Bl, all substan-
tially as described and
set forth.
Claim.—1. As an im-
proved article of man-
ufacture, a banket-muz-
zle consisting of a band
or support provided
with attaching mechan-
ism for attaching the
support to the head of a
horse, and a link apron
pendent from the rear
portion of such support
and adapted to extend
below the mouth of the
horse when in use, sub-
stantially as described.
2. In a blanket-muz-
zle, the combination,
with a nose-band and
detachable connections
on such band for con-
necting the band with a
headstall, of an apron pendent from the rear portion of such band and adapted to
extend below the mouth of the horse when in use, such apron being composed of
a plurality of interconnected links, substantially as described.
Claim.—In a device
for opening animals’
mouths, a standard pro-
vided with a bit, a hand-
lever and a rack, a trav-
eler provided with a bit,
a hand-lever and a re-
cess, a catch pivoted in
said recess and provided
with a handle, and a
spring for actuating said
latch, substantially as
and for the purposes set
forth.
Claim.—The combin-
ation, in a horse-boot,
of the strap 1, having
the pad 2 and padded
block, a flexible projec-
tion secured to said
block, consisting of a sin-
gle strip folded and hav-
ing its outer end cut
away so as to form the
ears 9, and said folds be-
ing stitched or secured
together as far as the
points 7a and unsecured
from such points to the
ends of the ears, so as to
form an opening between the folds, a spindle passing through the openings thus
formed in the ears, and a roller mounted on said pintle, substantially as set forth.
Claim.—The combin-
ation, with a boot, of a
strap secured thereto, an
angle-iron or device for
securing the boot to the
foot of the animal, and
an elastic connection in-
termediate the strap and
clip, substantially as de-
scribed.
2. The combination,
with the boot, of an ad-
justable strap secured
thereto, an angle iron or
clip for inserting between
the shoe and hoof of the
animal, and an elastic connection intermediate the strap or clip, such connection
being detachable.
Claim.—A pad of the
character described
comprising the two
padded flaps provided
with strips a a, the buck-
les C 0, the straps G G,
and a strap B, to which
said flaps are secured,
said strap adapted to en-
circle the horse, and a
buckle o for said strap,
substantially as de-
scribed.
				

## Figures and Tables

**Figure f1:**
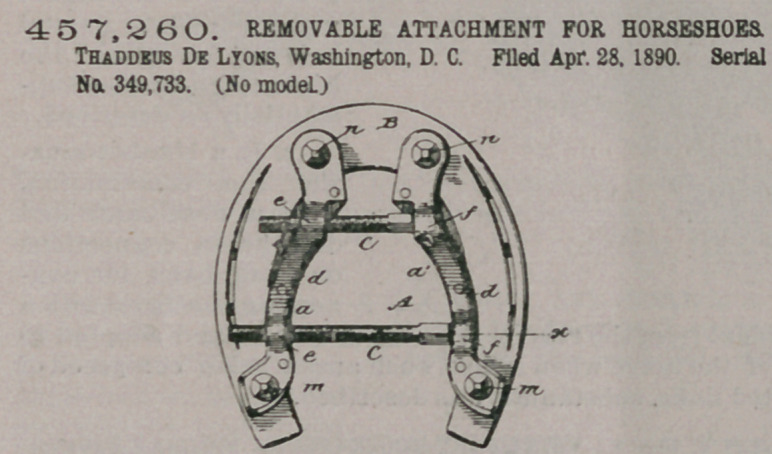


**Figure f2:**
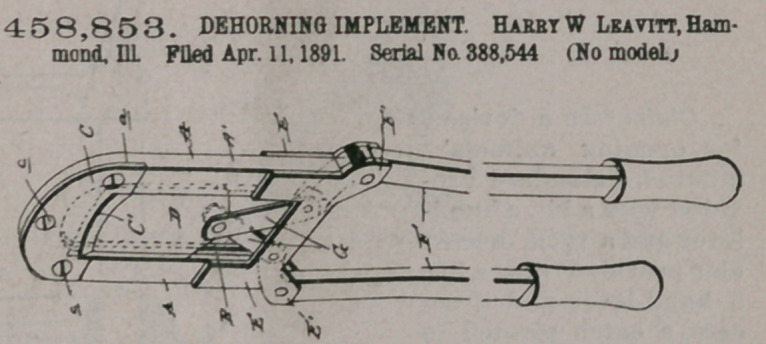


**Figure f3:**
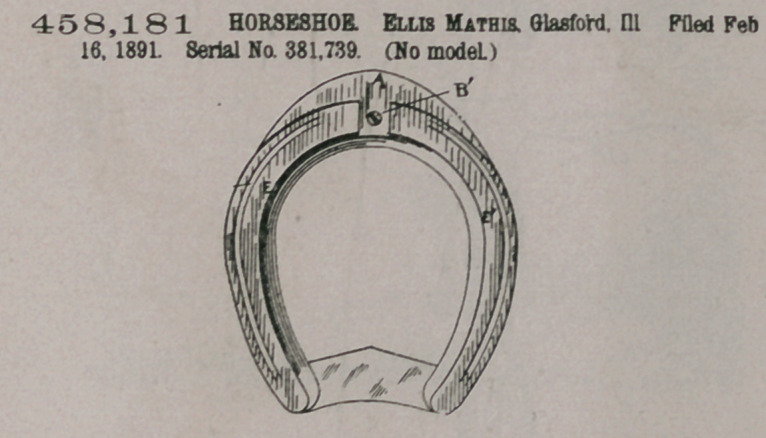


**Figure f4:**
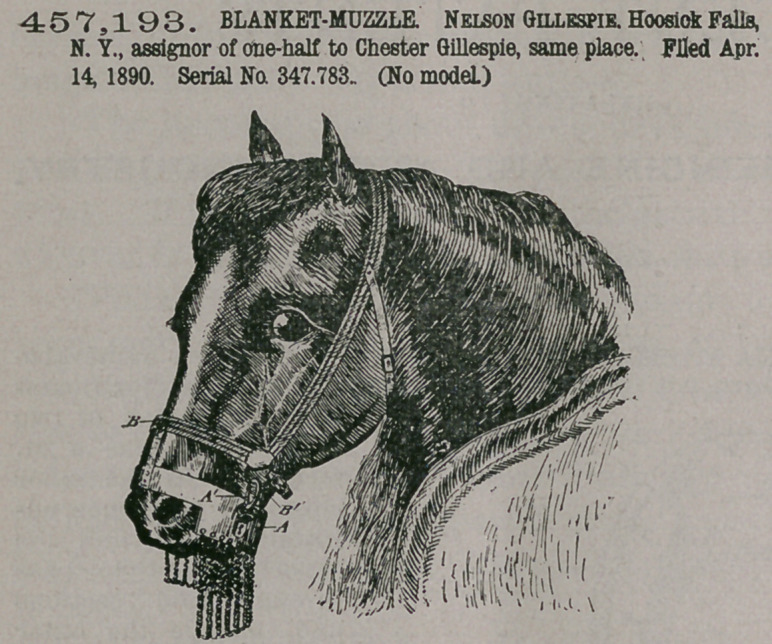


**Figure f5:**
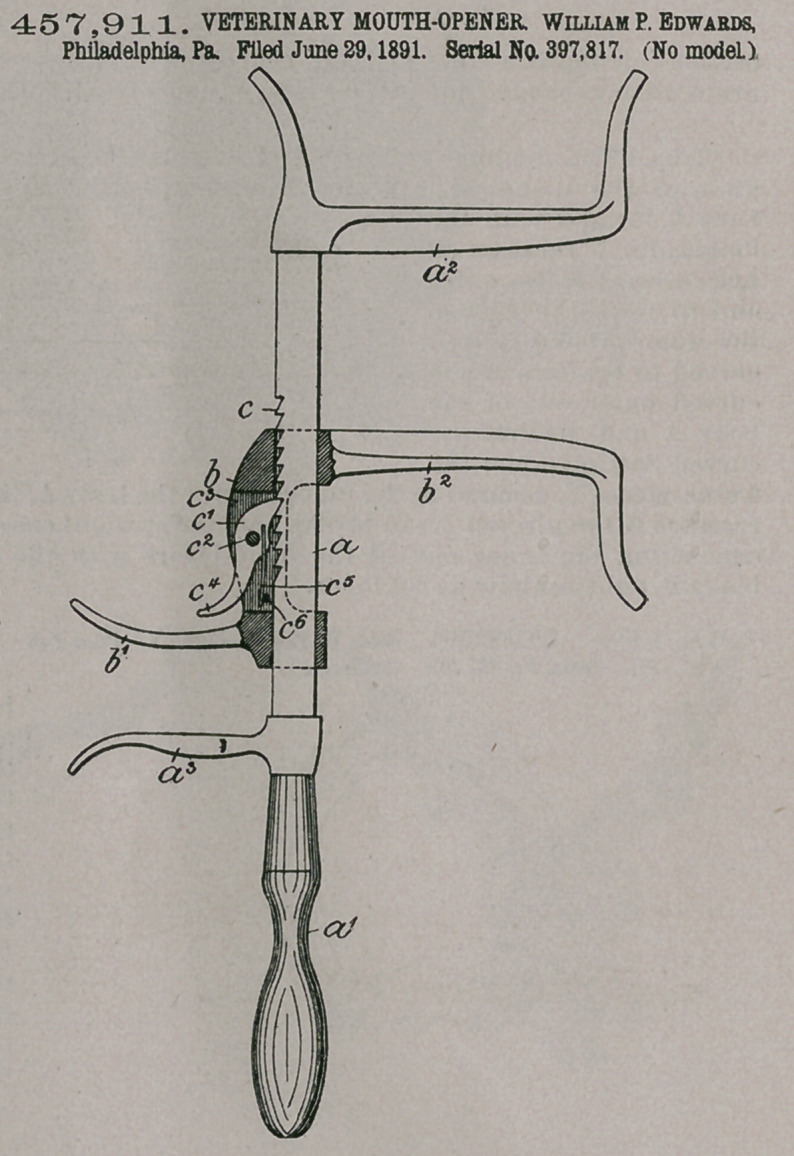


**Figure f6:**
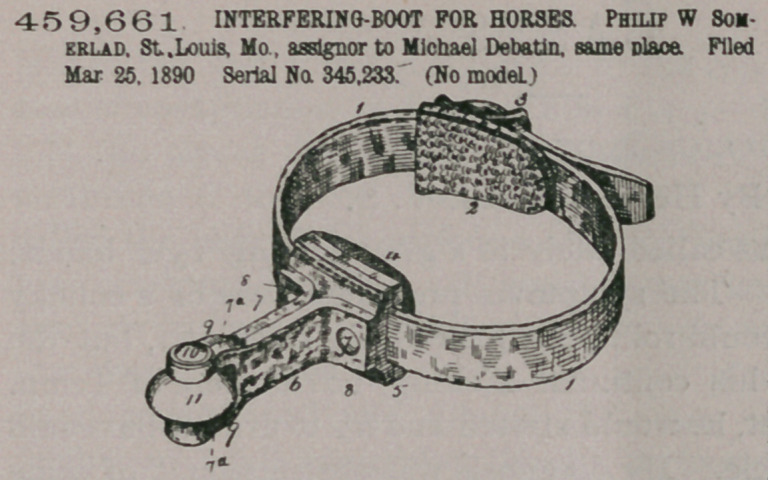


**Figure f7:**
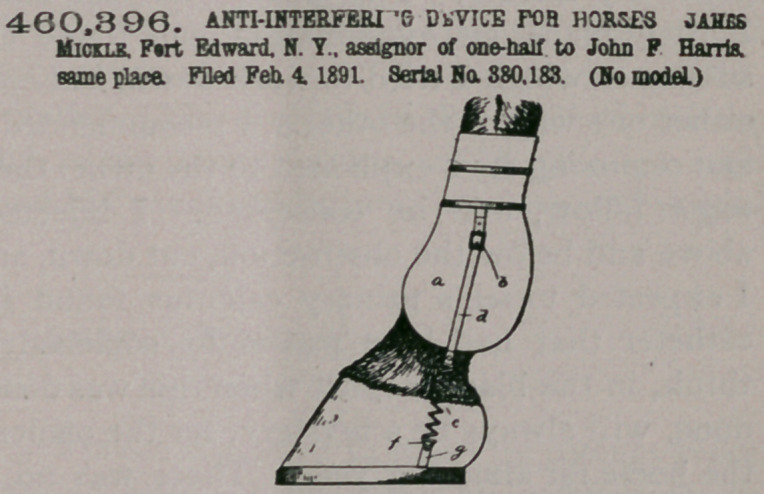


**Figure f8:**